# Roles of macrophages on ulcerative colitis and colitis-associated colorectal cancer

**DOI:** 10.3389/fimmu.2023.1103617

**Published:** 2023-03-16

**Authors:** Maorun Zhang, Xiaoping Li, Qi Zhang, Jiahua Yang, Gang Liu

**Affiliations:** Deparment of General Surgery, Tianjin Medical University General Hospital, Tianjin, China

**Keywords:** ulcerative colitis, colitis-associated colorectal cancer, macrophage infiltration, macrophage polarization, macrophage

## Abstract

Colitis-associated colorectal cancer is the most serious complication of ulcerative colitis. Long-term chronic inflammation increases the incidence of CAC in UC patients. Compared with sporadic colorectal cancer, CAC means multiple lesions, worse pathological type and worse prognosis. Macrophage is a kind of innate immune cell, which play an important role both in inflammatory response and tumor immunity. Macrophages are polarized into two phenotypes under different conditions: M1 and M2. In UC, enhanced macrophage infiltration produces a large number of inflammatory cytokines, which promote tumorigenesis of UC. M1 polarization has an anti-tumor effect after CAC formation, whereas M2 polarization promotes tumor growth. M2 polarization plays a tumor-promoting role. Some drugs have been shown to that prevent and treat CAC effectively by targeting macrophages.

## 1 Introduction

Ulcerative colitis (UC) is a chronic nonspecific intestinal inflammation caused by a variety of factors including heredity, immunity and environment exposure. Hemorrhagic diarrhea, abdominal spastic pain, and weight loss are the most common symptoms (1). In the past few decades, the incidence trend of UC is on the rise (2). Most ulcerative colitis patients only need medication to control their symptoms. When severe bleeding, obstruction or tumorigenesis occur, surgical treatment is required. Colitis-associated colorectal cancer (CAC) is the most serious complication of UC. The risk of tumorigenesis of UC includes long course of disease, wide range of lesions, and primary sclerosing cholangitis (3). Previous studies have shown that the incidence of colorectal cancer in patients with ulcerative colitis in Asia is 0.02%, 4.81% and 13.91% in 10 years, 20 years and 30 years, respectively (4). CAC is distinguished from sporadic colorectal cancer by the presence of multiple lesions, more serious pathological types, and a poor prognosis (5, 6). CAC and sporadic colorectal cancer(S-CRC) have different pathogenesis. S-CRC develops according to the sequence of adenoma–dysplasia–carcinoma, while CAC follows the sequence of inflammation–dysplasia–carcinoma. DNA damage caused by inflammation and reactive oxygen species promotes the occurrence of mutations. The mutations that occur in the development of CAC are similar to those of S-CRC, but the order of mutations is opposite. For example, in the development of CAC, the mutation of TP53 occurs in the early stage and the mutation of APC occurs in the late stage. In the process of sporadic colorectal cancer, TP53 mutation occurs in the late stage and APC mutation exists in the early stage (7). Despite accounting for a small proportion of colorectal cancer, CAC is still a leading cause of mortality and the reason for colectomy in UC patients (8).

The cause of UC tumorigenesis is not clearly understood. However, now most physician believe that it is related to the inflammatory microenvironment caused by intestinal inflammation (9). Inflammatory factors act on the intestinal mucosa, promoting the occurrence of tumor. Macrophages, as innate immune cells, contribute to the UC’s inflammatory response. Furthermore, macrophages play an important role in tumors. Therefore, it is critical to comprehend the role of macrophages in UC carcinogenesis and CAC progression. This article reviews the role of macrophages in the occurrence and progression of CAC.

## 2 Macrophage

Macrophage is an innate immune cell, most of which originate from monocytes (10). Recent research suggests that have monocytes may not be the only source of macrophages, and intestinal macrophages are derived from resident macrophages in the intestine. Macrophages secrete cytokines (11), remove cell fragments (12), kill pathogens (13), and are involved in inflammation (14), tissue repair (15), angiogenesis (16) and so on. Macrophages exist in many tissues of the body and play different roles in different tissues: alveolar macrophages in the lungs can maintain the stability of the environment in the alveoli (17), osteoclasts in bones have the ability of bone remodeling (18), and a large number of macrophages in the liver are called Kupffer cells which participate in immune response in liver (19, 20).

### 2.1 Basic biology of macrophage

Macrophages are white blood cells that exist in tissue. It is generally believed that macrophages are derived from monocytes, while monocytes are derived from the granulocyte-macrophage colony (GM-CFUc) forming unit in bone marrow. Monocytes migrate from blood to different tissues and form groups of cells with different functions, such as “inflammatory” monocytes and “resident” monocytes distinguished by the expression of cell surface marker CX3CR1 (21). Inflammatory monocytes have a short half-life and can differentiate into inflammatory macrophages and dendritic cells. Inflammatory monocytes may also be one of the sources of resident monocytes. Resident macrophages are usually produced by resident monocytes and sometimes by inflammatory monocytes. Resident macrophages and Foxp3^+^T cells play an important role in maintaining the stability of intestinal environment through the mechanism of IL-10 and TGF-β dependence. When intestinal inflammation occurs, inflammatory monocytes migrate to the intestinal tract and differentiate into dendritic cells and inflammatory macrophages, which can produce a variety of cytokines involved in inflammatory response (22). Inflammatory macrophages are usually activated as M1 phenotype, while resident macrophages are usually activated as M2 phenotype. This process, which is affected by a variety of complex factors, is known as polarization. The polarization of macrophages is dynamic and could be reversed under certain conditions. The polarized macrophages mainly include M1 activated by classical pathway and M2 activated by alternative pathway (23). M2 can be divided into four subtypes in response to various stimuli: M2a (24), M2b (25), M2c (26) and M2d (27).

Inflammatory cytokines, such as IFN- γ, TNF-α, and the microbial product LPS stimulate M1 polarization of macrophages (28). TLR2, TLR4, CD80, CD86, and CCR7 are among the surface receptors expressed by M1. And M1 secretes a variety of inflammatory cytokines and chemokines, including TNF-α, IL-6, IL-1, IL-12, ROS, CXCL9, CXCL10, CXCL11, CCL2, CCL3, CCL4 and CCL5 (29). Polarized M1 macrophages have increased antigen presentation ability (30). As a result, M1 shows strong inflammation, antibacterial and anti-tumor performance. Th-2 cytokines IL-4, IL-10 and IL-13 can stimulate M2 polarization of macrophages (31). Dectin-1, mannose receptor, scavenger receptor A/B, DC-SIGN (CD209) and CD163, CCR2, CXCR1, and CXCR2 expression in M2 macrophages. As the main member of tumor-associated macrophages TAMs, M2 has strong phagocytic ability, removes fragments and apoptotic cells, promote tissue repair, angiogenesis, suppresses immunity, and promotes tumor progression and metastasis (32).

### 2.2 Interaction between macrophages and other immune cells

The immune state of the body is maintained by the coordination of a variety of immune cells. The interaction between macrophages and other immune cells is complex and diverse, which can regulate the direction and intensity of immune response through a variety of ways and mechanisms, thus having different effects on the body.

#### 2.2.1 T cells

T cells are important immune cells, which participate in adaptive immunity. In tumor immunity, T cells can directly kill tumor cells and coordinate different anti-tumor immunity. As antigen presenting cells, macrophages affect the immune response of CD8+T cells by phagocytosis and degradation of antigens such as pathogens and tumor cells, or by regulating the expression of costimulatory molecules (33). In addition, macrophages can regulate the immune response of CD8+T cells by secreting cytokines and chemokines. M1 macrophages can promote the activation and proliferation of CD8+T cells and enhance their killing ability by releasing cytokines such as IL-12 and IL-18, thus clearing pathogens and tumor cells. M2 macrophages may inhibit the activation and proliferation of CD8+T cells and weaken their killing ability by releasing immunosuppressive factors such as IL-10, which may lead to immune tolerance or immune escape (34). M1 macrophages can secrete chemokines such as CXCL9/10 to attract CD8+T cells to the infection site and promote inflammatory response, while M2 macrophages can secrete chemokines such as CCL17 to attract Treg to the inflammatory site, thus inhibiting the inflammatory response (35, 36). In addition, Th1 cells secrete cytokines such as IFN- γ, which can stimulate M1 polarization of macrophages. And M1 macrophages can also secrete some chemokines, such as CXCL10 and CXCL11, which can attract and activate Th1 cells and enhance cellular immune response. Th2 cytokines such as IL-4 and IL-13 can stimulate M2 macrophage differentiation and activation which in turn can promote sustained Th2 T cell activation and survival (12). M1 macrophages activate signal transduction pathways such as NF- κB pathway and STAT3 pathway by secreting cytokines such as IL-1 β, IL-6 and IL-23, and then promote the differentiation and function of Th17 cells, thereby enhancing inflammatory response. M2 macrophages inhibit the differentiation and function of Th17 cells by producing anti-inflammatory factors such as IL-10 and TGF- β, thus reducing inflammatory response (37). Some studies have shown that IL-17 produced by Th17 can promote the differentiation and function of M1 macrophages, while inhibit the differentiation and function of M2 macrophages, thus enhancing the inflammatory response (38). However, other studies have also found that IL-17A can induce the differentiation and function of M2 macrophages, thus reducing the inflammatory response (39).

#### 2.2.2 B cells

B cells can secrete antibodies to participate in adaptive immunity. Macrophages can recognize and absorb antigens such as pathogens and present them to B cells, thus activating the immune response of B cells and promoting the production of antibodies (40). And M1 macrophages can activate B cells by secreting TNF- α, IL-6 and other cytokines, promote the proliferation and differentiation of B cells, and induce B cells to secrete antibodies such as IgM and IgG. On the other hand, M2 macrophages can secrete immunosuppressive factors such as IL-10 and TGF- β to inhibit the activation and differentiation of B cells, thus reducing the level of humoral immunity (41).

#### 2.2.3 NK cells

The interaction between M1 macrophages and NK cells usually helps to enhance cytotoxicity, while the interaction between M2 macrophages and NK cells may lead to immunosuppression. On the one hand, M1 macrophages can activate NK cells by secreting cytokines such as IL-12, IL-15, IL-18 and IFN-γ. These cytokines can promote the proliferation and differentiation of NK cells and enhance their cytotoxicity. On the other hand, the interaction between M2 macrophages and NK cells is relatively complex. Some studies have shown that M2 macrophages can inhibit the activity of NK cells by secreting cytokines such as IL-10. In addition, M2 macrophages can also inhibit the function of NK cells by expressing costimulatory molecules such as PD-L1 (42).

#### 2.2.4 ILCs

Unlike other lymphocytes, ILCs does not have T cell receptor or B cell receptor, so it does not participate in specific immune response. On the contrary, they play an important role in innate immunity. ILC is usually divided into three different subtypes: ILC1,ILC2 and ILC3, which play an important role in regulating immune response. M1 macrophages can secrete IL-12 and IL-18 to promote the differentiation and function of ILC1. ILC1 mainly produces IFN- γ and participates in antiviral and cytotoxic effects. M2 macrophages can secrete IL-33 to promote the differentiation and function of ILC2. ILC2 mainly produces cytokines such as IL-4, IL-5, IL-9 and IL-13, which are involved in anti-parasite infection, allergic reaction and tissue repair. Macrophages can secrete IL-23 to regulate the differentiation and function of ILC3. ILC3 mainly produces cytokines such as IL-17 and IL-22, and participates in antifungal and antibacterial effects (43, 44).

### 2.3 Roles of macrophage in UC

Skin and mucosa are the first line of immune defense of the body. In intestinal mucosa, macrophages and dendritic cells play an innate immune role, while B cells and T cells participate in adaptive immune response. Macrophages are important cells in chronic inflammation and the pathological processes. And macrophage infiltration is a sign of chronic inflammation. The polarization of macrophages is associated with some autoimmune diseases, such as UC (45) and systemic lupus erythematosus (46). Under physiological conditions, macrophages in the intestine phagocytize microorganisms and present antigens to activate T cells. Excessive activation of macrophages transforms physiological inflammation into pathological damage of intestinal mucosa. The number of macrophages increased significantly in active ulcerative colitis, suggesting that macrophages were involved in the occurrence and development of UC (47). And during the active phase of UC, most of the macrophages in the lamina propria of the intestinal wall are M1 phenotype. M1 breaks down tight junction proteins, destroys the epithelial barrier, induces apoptosis of epithelial cells, and leads to excessive inflammation (48). M1 plays a major role in intestinal inflammation, while M2 plays an antagonistic role which eliminates inflammation and promotes tissue healing. Previous studies have confirmed that increasing the proportion of M2 could reduce the symptoms of colitis in mice (49).

A variety of inflammatory cytokines secreted by macrophages play an important role in UC. IL-6 is an important mediator in inflammatory response, which directly participates in inflammatory response and corresponding injury process. IL-6 increases the permeability of epithelial cells, which promotes the infiltration of macrophages and aggravate the development of ulcerative colitis. Previous studies have shown that the level of IL-6 is positively correlated with the severity of intestinal inflammation (50). IL-18 is also highly expressed in serum and colon tissues of patients with ulcerative colitis. The application of IL-18 inhibitor relieves the symptoms of mouse colitis model and reduce the expression of many inflammatory factors (51). TNF-α regulates the NF-κB pathway by promoting IκBα degradation, NF-κB p65 phosphorylation and NF-κB nuclear transfer, which aggravates the injury of ulcerative colitis (52). Infliximab has been used as a TNF-α antagonist in the treatment of ulcerative colitis (53).

These cytokines not only play a role in the inflammatory response, but also participate in the tumorigenesis and progression of tumors. TNF- α is a kind of inflammatory cytokine with strong anti-tumor effect (54). Many kinds of immune cells, including macrophages, can produce TNF- α. It has been shown to promote tumor growth in chronic inflammatory diseases, although TNF- α has anti-tumor property. In chronic inflammation, by production of RONS, TNF- α promotes DNA damage caused by oxidative stress and promotes the tumorigenesis of CAC (55). And targeting TNF- α may prevent or reduce the tumorigenesis of CAC (56). Similarly, IL-6 can also promote early CAC tumorigenesis by inducing oxidative stress (57).

Interestingly, although TNF- α has anti-tumor effect, even some studies have shown that TNF- α can promote tumor cell apoptosis and inhibit tumor liver metastasis in colorectal cancer (58). However, more studies have found that TNF- α plays an important role in tumor progression. By stimulating epithelial-mesenchymal transformation, TNF- α can enhance the ability of invasion and metastasis of colon cancer cells (59). And TNF- α can promote angiogenesis by inducing human fibroblasts to secrete VEGF (60). TNF- α can also promote tumor lymphangiogenesis and lymphatic metastasis (61). In addition, TNF- α promote the progression and metastasis of CRC through NF- κ B pathway and induction of MACC1 (62, 63). By blocking TNF- α, the promotion of TNF- α on tumor can be reduced (64, 65).

## 3 Roles of macrophages on CAC

Macrophages have the dual functions of anti-tumor and promoting tumor in cancer. In view of the fact that M2 plays a promoting role in tumor development and metastasis, reducing the proportion of M2 polarization could be used as a way to treat tumor (66). On the other hand, M1 has the anti-tumor effect of promoting tumor immunity (67). The polarization of macrophages is affected by tumor microenvironment, which indicates that macrophage has the ability to become potential target for tumor therapy. In CAC, macrophages not only play a role in tumor immunity, but also play a role in the inflammatory environment before tumor formation. Overactivated macrophages not only aggravate inflammatory damage, but also promote tumorigenesis.

### 3.1 Macrophage infiltration aggravates inflammation and promotes tumorigenesis of UC

Inflammation is an important cause of carcinogenesis of UC. Macrophage infiltration is one of the characteristics of inflammation in UC. A large number of infiltrating macrophages aggravate the mucosal damage caused by inflammation and promote the transformation from inflammation to tumor. The infiltration of macrophages and the release of pro-inflammatory factors promote the carcinogenesis of epithelial cells.

CCL2 and CX3CL1 are important cytokines that induce macrophage infiltration. By promoting the expression of these cytokines, macrophage infiltration is enhanced and the occurrence and development of CAC is promoted. MUC1 is a kind of cell surface mucin which exists in intestinal epithelial cells and some leukocytes. MUC1 promotes the expression of CCL2 and mediates the recruitment and activation of macrophages. Activated macrophages secrete IL-6 and promote the occurrence and development of CAC through IL-6/STAT3 pathway (68). Glucocorticoid is a drug for the treatment of active UC. In an acute experimental UC mouse study, it was found that glucocorticoid increased the expression of CCL2 and CX3CL1, promoted macrophage infiltration and promoted the occurrence of UC and CAC by targeting mTOR signal of intestinal epithelial cells (69). Corticotrophin-releasing hormone receptor (CRHR1) belongs to the CRH family and is highly expressed in inflammatory tissues adjacent to CAC tumor tissue. CRHR1 promotes the recruitment of macrophages, secretes more inflammatory factors such as IL-1b, IL-6 and TNF- α, and promotes the early occurrence of CAC (70).

And inhibition of macrophage infiltration reduces the occurrence and development of inflammation and CAC. By inhibiting the expression of CCL2, GDC-0575, a CHK1 inhibitor, reduces macrophage infiltration in the mouse colon, which may inhibit the occurrence of CAC and colitis (71). Suppressor of AP‐1 (SAR1), a tumor suppressor, down-regulates the expression of p-STAT1 and STAT1 in CAC cells, inhibits the activation of MCP-1/CCR2 axis, reduces macrophage infiltration, which may inhibit the tumorigenesis of CAC (72). Serum amyloid A (SAA) is an evolutionarily conserved protein family associated with inflammation. It was found that in the CAC mouse model, SAA deficient mice showed a decrease in macrophage infiltration, which may inhibits tumorigenesis of CAC (73). Nerve injury inducer protein 1 (ninjurin1) is a homogenous cell surface adhesion molecule, which plays an important role in cell migration. Overexpression of Ninjurin1 in macrophages weakens the infiltration of macrophages by targeting FAK signaling pathway, thus may inhibiting the tumor progression of CAC (74). Diphenyleneiodonium (DPI) is an inhibitor of NADPH oxidase, which inhibits a variety of inflammatory responses. Low-dose DPI inhibits macrophage infiltration and migration, reduce the production of pro-inflammatory cytokines such as TNF- α and IL-6, reduce inflammatory response and may inhibit tumorigenesis (75). Estrogen receptor β (Erβ) reduce the occurrence and development of tumor by reducing macrophage infiltration and reducing the production of TNF-α (76). As a non-coding RNA, miRNA cannot encode proteins, but it can still exert its biological functions in a variety of physiological or pathological processes. A large number of previous studies have confirmed that miRNA plays an important role in inflammation and tumor (77, 78). Abdullah et al. found that overexpression of miR-132 inhibits macrophage infiltration and the production of proinflammatory cytokines, which may inhibit CAC (79).

**Figure 1:**
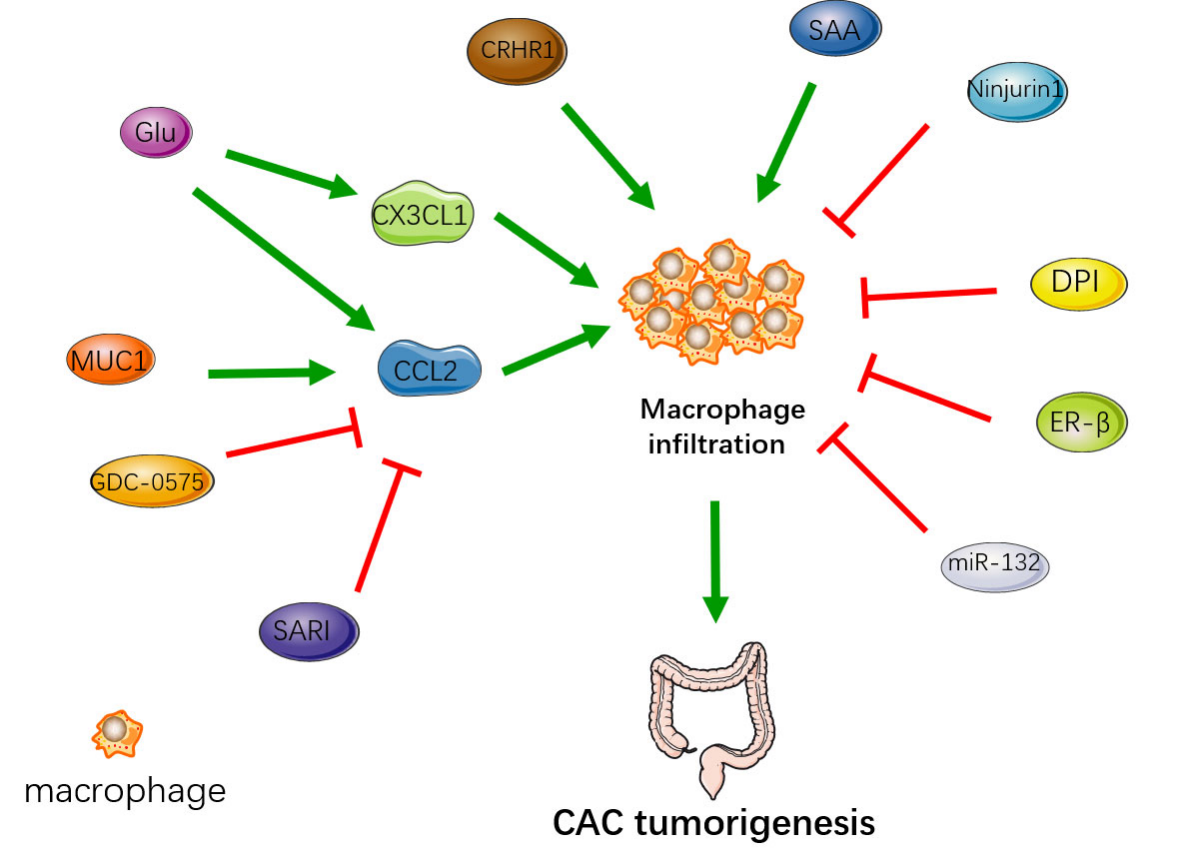
The regulation of intestinal macrophage infiltration affects the occurrence of CAC. MUC1, Glu, CRHR1 and SAA enhance macrophage infiltration to promote the tumorigenesis. SARI, GDC-0575, Ninjurin1, DPI, ER-β, miR-132 reduce macrophage infiltration to inhibit the tumorigenesis.

These factors that affect macrophage infiltration are quite pleiotropic and have numerous downstream targeting effects, so the impact on CAC may not be direct, and may not all be caused by macrophage infiltration. And the effect of macrophage infiltration on CAC mainly occurred in the early stage. The underlying mechanism may be the large amount of macrophage infiltration leads to more severe intestinal inflammation. A large number of cytokines produced by macrophages promote DNA damage and promote the process of inflammation-dysplasia-tumor. The regulation of intestinal macrophage infiltration affects the tumorigenesis of CAC (**Figure 1**). Therefore, early inhibition of macrophage infiltration may be of significance to not only reduce the inflammation of ulcerative colitis but also prevent the occurrence of CAC.

### 3.2 The influence of macrophage polarization on CAC

Macrophages express different phenotypes under the influence of different environments, which is called macrophage polarization (80). The polarized macrophages mainly include M1 activated by classical pathway and M2 activated by alternative pathway. M1 and M2 have different functions. M1 secretes inflammatory cytokines and enhances the inflammatory response, but it has the anticancer effect of enhancing tumor immunity in tumor tissues. M2 promotes tissue repair and has anti-inflammatory effect, but it is considered to inhibit tumor immunity in tumor tissue. This means that macrophage polarization can not only affect the occurrence of CAC by regulating inflammation, but also directly affect CAC by regulating tumor microenvironment.

Excessive M1 polarization aggravates the inflammatory symptoms of the UC, promotes mutation involvement and provides an inflammatory environment suitable for tumorigenesis. M2 antagonize the pro-inflammatory effect of M1 and inhibit tumorigenesis. Low-dose DPI inhibited the classical activation pathway of macrophages and the level of M1 by down-regulating the pathways of STAT3, NF-κB and ERK, and inhibited the tumorigenesis of CAC in the early stage (75). MiR-146b targets MyD88 and IRAK1, regulates TLR4 pathway, inhibits M1 activation, and then reduces inflammation and inhibits the tumorigenesis of CAC (81). In the early stage of CAC, xanthine oxidoreductase (XOR) mediated M1 macrophage polarization, which promoted the tumorigenesis of CAC (82). In macrophage, Fibrinogen-like protein 2 (Fgl2) deficiency promotes M1 polarization and inhibit M2 polarization, which promote the tumorigenesis of CAC (83).

However, inflammation is not the only factor affecting the tumorigenesis of UC. M2 macrophages in tumor microenvironment also have the effect of pro-tumor. In myofibroblasts, MyD88 secretes OPN through TLR pathway, binds to CD44 and αvβ3 on macrophages, promotes M2 polarization through STAT3 and PPARγ pathway, which promotes the tumorigenesis of CAC (84). And a study found that the loss of SLC7A2 enhanced M2 polarization to promote the tumorigenesis of CAC (85).

**Figure 2:**
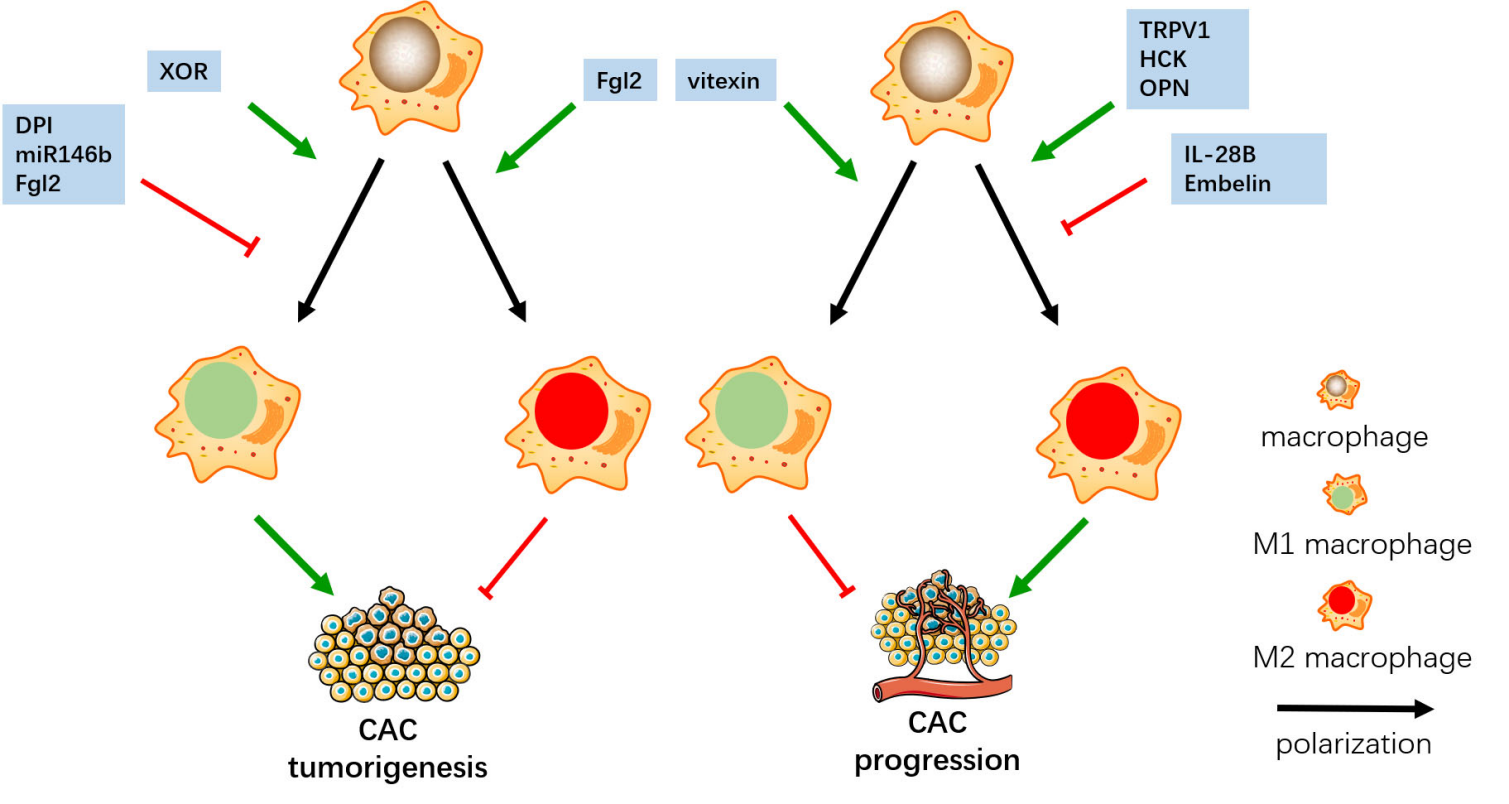
In the early stage, DPI, miR146b and Fgl2 inhibit M1 polarization, and Fgl2 promote M2 polarization, which both inhibit the tumorigenesis of CAC. XOR promotes M1 polarization and enhance the tumor progression of CAC. In the later stage, vitexin promotes M1 polarization, IL-28B and Embelin inhibit M2 polarization, which both inhibit CAC progress. TRPV1, HCK and OPN promote M2 polarization and enhance the tumor progression of CAC.

After tumor formation, M1 plays an anti-tumor role through direct tumoricidal mechanisms or by promoting CD8 cytotoxic T cell and NK cell killing of tumor cells in later phases of CAC progression. M2 promotes tumor immune escape and enhanced tumor progression, especially invasion and metastasis. In a study on vitexin in CAC mice, vitexin inhibited M1 polarization in inflammatory tissue, but promoted M1 polarization in tumor tissue, which reduces tumorigenesis, inhibited tumor progression, and reduced tumor load (86). Transient receptor potential vanilloid 1 (TRPV1) is an ion channel expressed in a variety of immune cells. In the early stage of CAC, TRPV1 directly stimulates M1 polarization and promotes tumorigenesis. After that, TRPV1 regulates the Calcineurin/NFATc2 pathway to increase the phenotype of Th2, enhance M2 polarization and promote tumor progression (87). IL-28B inhibits M2 polarization of macrophages through STAT3 and JNK signaling pathways, which reduces the expression of M2 cytokines Arg-1 and TGF-β, to inhibit the tumor progression of CAC (88). Hematopoietic cell kinase (HCK) is one of nine non-receptor tyrosine kinases in the SRC family, which has the function of regulating innate immunity. HCK promotes M2 polarization which promotes the tumor progression of CAC (89) (**Figure 2**).

Tumor immunity is an important factor affecting tumor progression. The main reason for the effect of macrophage polarization on CAC is that polarized M1 and M2 play different functions in tumor immunity.

M1 produces ROS, which can directly kill tumor cells and inhibit tumor growth. M1 macrophages can produce a series of pro-inflammatory cytokines, including TNF- α, IL-1 β, IL-6, IL-12. These cytokines can stimulate the activity of immune cells such as NK cells and CD8+T cells and enhance their ability to attack tumor cells (90). In addition, M1 macrophages can activate tumor-specific T cells through antigen presentation and mature DC cells, thus promoting anti-tumor immune response (91). Recent studies have shown that M1 macrophages can also enhance anti-tumor immune response by inhibiting immunosuppressive cells in tumor microenvironment, including Tregs and MDSCs (92).M2 cells play an important role in different stages of tumor progression. In the early stage of tumor, M2 macrophages are mainly involved in tumor growth and angiogenesis. They secrete a variety of growth factors and cytokines, including VEGF, TGF- β, EGF, which promote tumor cell proliferation and neovascularization. In the middle stage of the tumor, M2 macrophages mainly play an immunosuppressive role. They can inhibit the activity of tumor immune cells and reduce the immune recognition and killing of tumor cells by secreting a variety of cytokines and surface molecules, including IL-10, TGF- β, PD-L1. In the late stage of tumor, M2 macrophages are mainly involved in tumor invasion and metastasis. By secreting a variety of proteases and lysosomal enzymes, they can promote tumor cell infiltration and invasion of surrounding tissues, and promote tumor cell metastasis and distant metastasis (36, 93–95). In general, M2 macrophages have complex mechanisms in the process of tumor growth and development, which can not only promote tumor growth and angiogenesis, but also inhibit the activity of tumor immune cells and promote tumor invasion and metastasis. Tumor cells also produce cytokines that affect the differentiation and function of immune cells. Some studies have shown that G-CSF released by tumor cells can activate MDSCs, thus forming an immune microenvironment suitable for tumor progression. In mechanism, MDSCs expresses high levels of inhibitory receptors and ligands, such as PD-L1, B7-H1 and CTLA-4, which can bind to T cell activation signal molecules (such as CD28), thus inhibiting T cell activation and proliferation. MDSCs can produce a variety of immunosuppressive molecules, including ROS, TGF- β, IL-10. These molecules can directly or indirectly inhibit the function and proliferation of T cells. MDSCs can promote T cell apoptosis through a variety of mechanisms, including secreting apoptosis-inducing molecules, expressing apoptosis-inducing receptors, inhibiting survival signals (96–98). These production of factors by MDSC and M2 macrophages provide a productive microenvironment tumor invasion and metastasis during tumor progression to malignant carcinoma stage tumors, including CAC.

### 3.3 The mechanism of macrophages affecting CAC

In addition to infiltration of a large number of macrophage and break of the balance of macrophage polarization, macrophages also affect CAC through some other mechanisms such as regulating the secretion of inflammatory cytokines or tumor-promoting factors and targeting NF-κB pathway in macrophages.

Pou3f1 is a member of POU family and participates in cell apoptosis and immune response (99, 100). Nuclear factor of activated T cell 3 (Nfatc3) targets Pou3f1 to increase its expression, while Pou3f1 can promote inflammation in macrophages. Knockout of Pou3f1 decreased the levels of pro-inflammatory cytokines such as IL-1β, IL-6 and TNF-α, and inhibited the tumorigenesis of CAC (101). G protein-coupled receptor 35 (GPR35) is a soliton G protein-coupled receptor that interacts with sodium-potassium pumps. In macrophages, GPR35 promotes macrophages to release angiogenic factors and promote angiogenesis and tumor metastasis through the activation of Na/k-ATPase and non-receptor tyrosine kinase SRC (102). Dana et al. found that in macrophages, EGFR signal activates M2 polarization through STAT6, which promotes angiogenesis and CAC progression. Interestingly, in this study, M1 polarization is also activated by EGFR signals *via* NF-κB. The promotion of angiogenesis and tumor metastasis is due to the joint action of M1 and M2 to produce angiogenic factors CXCL1 and VEGF (103). Macrophage scavenger receptor A1 (SR-A1) is a pattern recognition receptor, which is mainly expressed in macrophages. In macrophages, SR-A1 inhibits the classical and non-classical activation of NF-κB signal through TRAF6 and TRAF3, respectively, thus inhibiting the occurrence of colitis and CAC (104). Elongation factor Tu GTP binding domain containing 2 (EFTUD2) is a mRNA splicing protein that could promote the occurrence of CAC by regulating the inflammatory response of macrophages. In macrophages, EFTUD2 deletion reduces the secretion of inflammatory factors and inhibits the occurrence of CAC by inhibiting the activation of TLR4-NF-κB pathway (105). MiRNA26a is overexpressed in macrophages, targeting IL-6, TLR3 and PKCσ, inhibiting the activation of NF-κB/STAT3 pathway and inhibiting the occurrence and development of CAC (106). M1 promotes the expression of TNF-related apoptosis-inducing ligands (TRAIL) in adipose tissue-derived stem cells (ASCs), while ASCs induce apoptosis of CD133+ tumor cells and reduce the number of M2 through TRAIL, which inhibits the progress of CAC (107).

The discovery of these mechanisms not only gives us a deep understanding of the role of macrophages in CAC, but also provides a new direction for the treatment of CAC by targeting these molecular pathways. At present, some potential drugs for the treatment of CAC, especially traditional Chinese medicine, play a role by affecting macrophage infiltration or polarization. Some drugs that directly target these molecular pathways are highly anticipated.

## 4 Drugs that regulate CAC through macrophages

5-ASA is thought to reduce the risk of CAC in patients with UC, which may be related to its effect on controlling inflammation (108). In view of the important role of macrophages in CAC, some drugs can prevent and treat CAC by targeting macrophages (**Table 1**).

**Table 1:** Drugs that regulate CAC through macrophages.

Drug	Effect on macrophages	Function on CAC	References
DI	Inhibit macrophage infiltration	Inhibit tumorigenesis	(109)
cetuximab	Inhibit M2 polarization	Inhibit tumorigenesis and angiogenesis	(103, 110)
DPI (ultralow dose)	inhibit M1 polarization and macrophage infiltration	inhibit tumorigenesis	(75)
OTSSP167	inhibit M1 polarization and macrophage infiltration	inhibit tumorigenesis	(111)
mannose	inhibit M2 polarization	inhibit tumorigenesis	(112)
Embelin	inhibit M2 polarization	Inhibit angiogenesis	(85)
YYFZBJS	inhibit M2 polarization	Inhibit tumorigenesis	(113)
GLP	inhibit macrophage infiltration	inhbit tumorigenesis	(114)
SYD	inhibit macrophage infiltration	Inhibit tumorigenesis	(115)
YTE-17	Inhibit M2 polarization	Inhibit tumorigenesis	(116)
triptolide	Inhibit M2 polarization	Inhibit tumor growth	(117, 118)
ISL	Inhibit M2 polarization	inhibit tumorigenesis	(119)
Vitexin	Inhibit M1 polarization (Inflammatory tissue) promote M1 polarization (tumor tissue)	Inhibit tumorigenesis Inhibit tumor growth	(86)
DHA	Inhibit macrophage infiltration	Inhibit tumorigenesis	(120)
corylin	inhibit M1 polarization	Inhibit tumorigenesis	(121)

Dimethyl itaconate (DI) inhibits the secretion of IL-1 β, thus reduces the production of CCL2, restricts the recruitment of macrophages by the combination of CCL2 and CCR2, which inhibits CAC (109). EGFR signaling pathway plays an important role in the growth and metastasis of colorectal cancer. However, most of the studies focused on the colon epithelial cells. It has been found that EGFR in macrophages can regulate macrophage polarization and promote the tumorigenesis and angiogenesis of CAC. Targeting EGFR with cetuximab eliminates the cancer promoting effect of macrophages (103, 110). Ultra-low dose of DPI reduces the M1 polarization and macrophage infiltration of macrophages, and then inhibit the tumorigenesis and tumor progression of CAC. But it cannot kill CAC tumor cells directly (75). OTSSP167 inhibits macrophage infiltration and M1 polarization by targeting Maternal embryonic leucine zipper kinase (MELK), thereby inhibiting the tumorigenesis of CAC (111). Mannose inhibits M2 polarization to inhibit tumorigenesis of CAC (112). Embelin is a small molecule inhibitor of X-junction apoptotic protein inhibitor with anti-tumor effect. In the late stage of CAC, embelin reduce M2 polarization to reduce angiogenesis and MMP expression, which inhibits tumor metastasis (122).

Many traditional Chinese medicines have definite therapeutic effects on UC. Some traditional Chinese medicines or their active components regulate the occurrence and progression of CAC by targeting macrophages. Yi-Yi-Fu-Zi-Bai-Jiang-San (YYFZBJS) is a traditional Chinese medicine, and its extract inhibits the imbalance of enterotoxigenic bacteroid fragilis (ETBF) in the intestinal tract. The imbalance of ETBF stimulates STAT3-mediated polarization of M2 macrophages to promote malignant transformation of adenomas, thus promoting the occurrence and development of CAC. By inhibiting ETBF imbalance, YYFZBJS inhibits M2 polarization and plays an antitumor role in CAC (113). Ganoderma lucidum polysaccharide (GLP) is a component of Ganoderma lucidum, which reduce intestinal macrophage infiltration, down-regulate the expression of inflammatory factors such as IL-1 β, and attenuate the tumorigenesis of CAC (114). Shaoyao decoction (SYD), as a traditional Chinese medicine, also inhibit the proliferation of macrophages, reduce the expression of NF-κB and cytokines IL-1 β, IL-6, TNF-α, which inhibits the tumorigenesis of CAC. In addition, SYD down-regulates EMT and inhibits CAC by inhibiting the expression of cytokines (115). YTE17 is the active fraction of Garcinia yunnanensis. In CAC mouse model, YTE17 suppresses M2 polarization by down-regulating JNK, STAT3 and ERK signal pathways, thus inhibiting the tumorigenesis of CAC (116). Triptolide is the active ingredient of Tripterygium wilfordii, which has the effects of suppressing immunity and anti-inflammation. Triptolide inhibits M2 polarization of macrophages and reduce the secretion of anti-inflammatory cytokines. By inhibiting the promoting effect of M2 on the growth of CAC tumor, it plays an anti-tumor effect (117, 118). Isoliquiritigenin (ISL) is a flavonoid extracted from licorice and has anti-inflammatory effects. In CAC mouse model, ISL inhibits M2 polarization through cox-2/PGE2 pathway and IL-6/STAT3 pathway, and then prevents against tumorigenesis of CAC (119). Vitexin is the active ingredient of many traditional Chinese herbal medicines, which is found in hawthorn, mung bean and other traditional Chinese medicines. It has antioxidant, anti-inflammatory and antitumor effects. In the CAC mice, the M1 in the inflammatory tissues adjacent to the tumor of the mice treated with vitexin decreased and M2 increased. In tumor tissues, vitexin enhanced M1 polarization of macrophages. The reduction of inflammation of inflammatory tissue inhibits the occurrence of tumor. M1 polarization in tumor tissue promotes tumor immunity and reduces tumor burden (86). Dihydroartemisinin (DHA) is an active metabolite of artemisinin compounds, which could inhibit inflammation and tumor progression. At the early stage of CAC formation, DHA inhibits macrophage activation, reduces macrophage infiltration which inhibits the occurrence of CAC by inhibiting TLR4 signal. And in the late stage of CAC, DHA inhibited tumor growth by enhancing cell cycle arrest and apoptosis in tumor cells (120). Corylin is a natural polyphenol compound, which has antioxidant effect. It was found that corylin inhibits M1 polarization so that inhibits the tumorigenesis of CAC (121).

It is worth noting that these drugs mentioned above are in the preclinical animal model stage. Further human clinical trials are needed to determine whether it can be transferred to humans. In spite of this, we still have great expectations for targeted macrophage therapy for CAC.

In addition to drugs, diet also affects CAC by regulating macrophages. β-carotene (BC) has the ability of antioxidation. Previous study have found that drinking carrot juice rich in β-carotene reduces cell DNA damage (123). In addition, BC inhibits the growth of human leukemia and colon cancer by regulating NF-κB signal pathway (124). In CAC, BC inhibits tumor growth by inhibiting M2 polarization of macrophages (125).

Obesity is a risk factor for colorectal cancer. In obese mice model, IL-6 induces the expression of chemokine CCL-20 in M2, recruits immune cells expressing CCR-6, and promotes the progress of CAC (126). A study on western diet found that the western diet with high fat and low fiber promoted the infiltration of macrophages in colon tissue of mice, aggravated intestinal inflammation and promoted the development of CAC (127). Metformin combined with probiotics seems to inhibit the infiltration of macrophages and the destruction of intestinal epithelial barrier caused by western diet, thus inhibiting the occurrence of CAC (128).

## 5 Conclusion

The development from UC to CAC includes two processes: inflammation and tumor. Macrophages are also involved in both inflammation and tumor. Obviously, in the inflammatory stage of UC, excessive macrophage infiltration aggravates the damage of inflammation and promotes the occurrence of CAC. However, macrophages polarize into M1 macrophages and M2 macrophages, which play different roles in inflammation and tumor. M1 macrophages have the effects of promoting inflammation and antitumor, while M2 macrophages have the effects of anti-inflammation and promoting cancer. Before the tumor forms, M1 plays a pro-inflammatory role in promoting the tumorigenesis. After the formation of CAC, M1 polarization inhibits the progression of CAC by tumor immunity, while M2 promotes tumor progression and metastasis. Interestingly, some studies have found that M2 not only promotes tumor progression, but also increases the incidence of CAC (84, 85). This may be because M2 play a tumor-promoting role in the early stage of tumor formation.

Some drugs, especially traditional Chinese medicine, have been confirmed that play a role in the prevention and treatment of CAC by targeting macrophages. In the future, there is still great potential and possibility to prevent the occurrence of CAC and improve the prognosis of CAC patients by targeting macrophages.

## Author contributions

GL provided direction and guidance throughout the preparation of this manuscript. MZ and XL wrote and edited the manuscript. QZ reviewed and made significant revisions to the manuscript. JY collected and prepared the related papers. All authors contributed to the article and approved the submitted version.

## Glossary

**Table:**

ASCs	adipose tissue-derived stem cells
BC	β-carotene
CAC	Colitis-associated colorectal cancer
CRHR1	Corticotrophin-releasing hormone receptor
DHA	Dihydroartemisinin
DPI	Diphenyleneiodonium
EFTUD2	Elongation factor Tu GTP binding domain containing 2
Erβ	Estrogen receptor β
ETBF	enterotoxigenic bacteroid fragilis
Fgl2	Fibrinogen-like protein 2
GLP	Ganoderma lucidum polysaccharide
GPR35	G protein-coupled receptor 35
HCK	Hematopoietic cell kinase
ISL	Isoliquiritigenin
MELK	Maternal embryonic leucine zipper kinase
Nfatc3	Nuclear factor of activated T cell 3
SAA	Serum amyloid A
SAR1	Suppressor of AP‐1
SR-A1	scavenger receptor A1
SYD	Shaoyao decoction
TRAIL	TNF-related apoptosis-inducing ligands
TRPV1	Transient receptor potential vanilloid 1
UC	Ulcerative colitis
XOR	xanthine oxidoreductase
YYFZBJS	Yi-Yi-Fu-Zi-Bai-Jiang-San
